# Compound impacts from droughts and structural vulnerability on human mobility

**DOI:** 10.1016/j.isci.2022.105491

**Published:** 2022-11-23

**Authors:** Lisa Thalheimer, Nicolas Choquette-Levy, Filiz Garip

**Affiliations:** 1 School of Public and International Affairs, Princeton University, Princeton, NJ, USA; 2 Department of Sociology, Princeton University, Princeton, NJ, USA

**Keywords:** Climatology, Social sciences, Human Geography

## Abstract

Extreme dry events already disrupt populations’ ability to migrate. In a warming climate, compound drought events could amplify vulnerability and drive forced migration. Here, we contribute the first multi-method research design on societal impacts from compound drought events. We show how mobility patterns are shaped by the intersection of drought and social vulnerability factors in three drought-prone countries – Madagascar, Nepal, and Mexico. We find that internal migration in agricultural communities in Mexico increased by 14 to 24 basis points from 1991 to 2018 and will prospectively increase by 2 to 15 basis points in Nepal in case of a compound drought event in 2025. We show that consecutive drought events exacerbate structural vulnerabilities, limiting migrants’ adaptation options, including long-range migration. We conclude that the additional social pre-conditions, e.g., social isolation and lack of accurate information, ultimately limit migration as an adaptation option for households vulnerable to compound drought events.

## Highlights

• Compound droughts strongly affect internal migration in agricultural communities
• Mixed methods unveil how climate and vulnerability compound to shape migration outcomes
• In Mexico, within country migration increased by 14–24 basis points (1991–2018)
• In Nepal, a compound drought in 2025 could increase migration by 2–15 basis points

## Graphical abstract

**Figure:**
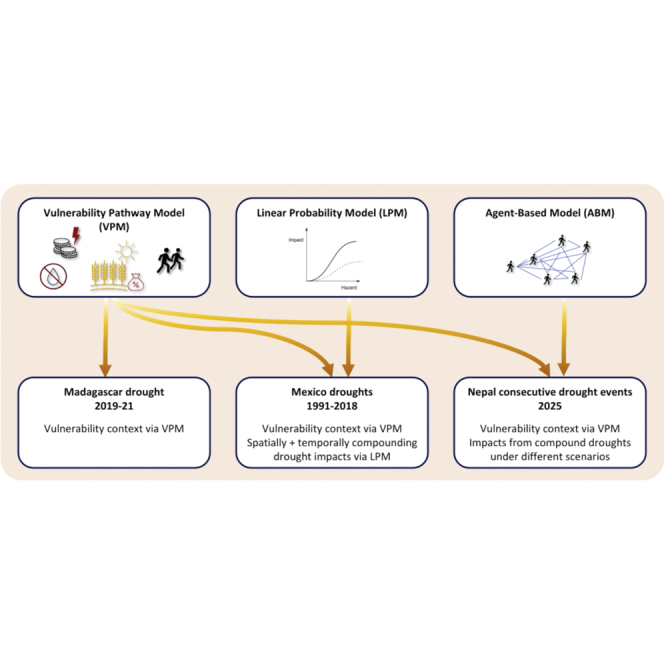

## Introduction

Extreme dry weather (potentially exacerbated by anthropogenic climate change) is already affecting those most vulnerable to a warming climate, among others, migrants and displaced populations.^1^ Pre-existing components of vulnerability contribute to the exposure of migrants and climate change is anticipated to aggravate these exposure levels.^2^ With additional climate change inputs, the population exposure to compound droughts is likely to be amplified through increases in El Nino–Southern Oscillation variability, paving the way to adverse compound socio-economic impacts.^3^ Compound events (CEs) which are the result of a combination of factors rather than one single issue, may stretch a population’s adaptive capacity, increasing risks of displacement and food insecurity.^4^^,^^5^^,^^6^ In order to limit societal impacts from CEs, the individual and compounding drivers of vulnerability need to be understood. Only recently, researchers have engaged in modeling societal outcomes of CEs with evidence on socio-economic impacts remaining limited and fragmented.^7^^,^^8^^,^^9^ The growing socio-economic importance of increased drought risks raises questions about the impacts from CEs on vulnerability components (see section 2).

We address this gap by conducting a multi-evidence, multi-location study on the interaction of drought impacts and different components of vulnerability in the drought-prone areas of Madagascar, Mexico, and Nepal. We combine a qualitative vulnerability pathway model (VPM) with quantitative evidence from a linear probability model (LPM) and with an agent-based model (ABM). We draw on large-scale survey data in Mexico and an agent-based model calibrated with socioeconomic and climate data from Nepal, and qualitative data from existing literature in Madagascar. We show how vulnerability components compound with drought risk to carve out differences in the effects on human mobility outcomes in the three study locations.

We contribute to two strands of the literature: First, our work is related to studies under the umbrella of climate change impacts,^10^^,^^11^^,^^12^ and more specifically to studies on the socio-economic and environmental determinants of drought vulnerability.^13^^,^^14^ We also expand on the literature on socio-economic impacts from CEs literature by assessing the impacts of compound droughts and structural vulnerability pre-conditions on human mobility.^15^^,^^16^^,^^17^^,^^18^ Second, we expand on the empirical evidence on the determinants of climate change impacts for vulnerable internal migrants in the agriculture sector.^19^^,^^20^^,^^21^^,^^22^ In the discussion section of our results, we will also refer to the closely related literature on extreme event attribution.^23^^,^^24^

By analyzing how drought vulnerability components vary spatio-temporally, we shed light on the relationships between drought exposure and human mobility responses, one of several examples for adaptive strategies that may be deployed by subsistence farmers.^25^^,^^26^^,^^27^^,^^28^^,^^29^ Examining this issue is crucial for designing policy measures and metrics that can strengthen resilience to CEs, particularly among vulnerable communities.

On the determinants of drought adaptation, existing studies come to ambiguous conclusions regarding the role played by migration. In a review on environmental factors influencing migration, Cattaneo et al.^30^ demonstrate that the range and accessibility of alternative non-migration adaptation options that are less cost-intensive or disruptive to livelihoods influence the decisions to migrate or not. Higher rural-to-urban migration has been observed in Mexico and Senegal after the onset of a drought event.^31^^,^^32^ A common response to drought is an increase in short-distance, temporal migration in low-income countries.^27^^,^^33^^,^^34^ Relatedly, Mueller et al.^35^ and Dillon et al.^36^ demonstrate that extreme heat is a strong predictor of male rural-urban migration in Pakistan and Nigeria, respectively, suggesting that the compound shocks of heat waves and drought on agricultural income may be a significant migration push factor. In a meta-analysis of empirical studies on the climate-migration literature, Hoffmann et al.^37^ find conflicting directional effects of climate variables on both internal and international migration flows. The seemingly contrary findings in this body of literature further highlight the importance of analyzing the impacts of drought on migration in the context of CEs and pre-conditions that may differ between geographic contexts.

Compared to existing studies, our multi-method setting allows us to uniquely disentangle the effects of vulnerability components and subsequently quantify the impacts of compound drought events. Such enhanced empirical evidence is relevant to better understand patterns of drought-related human mobility and ultimately, for an evidence-based design of policy measures and anticipatory funding mechanisms, e.g., forecast-based financing programs.^38^

### Defining vulnerability and compound drought events

In line with McLeman et al.,^17^ we define vulnerability as a function of hazard and exposure that affects human mobility outcomes. In our context, components of vulnerability refer to contextual vulnerability,^39^ in particular structural vulnerabilities, exposure to extreme dry events, adaptive capacity, which will be defined in the following. Structural vulnerabilities include structural underinvestment, limited livelihood options and poverty. Hazard refers to the extent, intensity, and frequency of drought events; exposure describes the populations affected by drought in a region. Adaptive capacity is a key element in vulnerability analysis,^40^ which refers to a household’s ability to further adapt to extreme events, in our context droughts.

We define compound drought events (CE) as the combination of multiple physical and social drivers and/or hazards that contribute to societal or environmental risk, adopting the definition and explanation introduced by Zscheischler et al.^6^^,^^41^ In this context, drivers include vulnerabilities that may spatially and temporally occur over multiple scales.^16^ We focus on drought CEs, that is, the co-occurrence and intersection of multiple vulnerability drivers and/or drought hazards in the same geographical region within Madagascar, Mexico and Nepal (Table 1).

**Table 1:** Overview on compound drought events, methods, and methodological contributions

Compound drought events and location	Method	Contribution	Pitfalls	Mobility type	Data
Drought 2019-2021, Tropical storms 2022 in Madagascar	Vulnerability pathway model (VPM)	Overview on vulnerabilities driving a risk factor related to human mobility	Visually excessive, contribution of vulnerabilities is difficult to quantify	Internal labor migration	Qualitative data: - extreme event attribution study (Harrington et al., 2021)^23^ - reports and estimates from international organizations (FEWS NET, 2020a,b)^42^^,^^49^; (BBC, 2021)^43^; (Walton et al., 2021)^46^; Khamis et al., 2021^47^; Nchanji & Lutomia, 2021^48^
Extreme dry months during corn season in Mexico (1980-1990)	Linear probability model (LPM)	Effect of structurally, spatially, and temporally compounding events on mobility outcomes	Heteroskedasticity predicted probabilities might be unbounded which complicates their interpretation	Internal migration	Quantitative data: - Mexican Migration Project (1991-2018) (MMP 2019)^75^ - Gridded daily weather data from NASA Earth Observing System Data and Information System (Thornton et al. 2020)^76^
Hypothetical drought(2025), hypothetical spike in temperatures (2022-2025), Social preconditions in Nepal	Agent-based model (ABM)	Interaction of mechanisms by which compound events and pre- conditions drive mobility outcomes	Requires Assumptions about future climate and socio-economic conditions; Model requires Some Abstraction from real-world Complexity	Internal, international Migration	Qualitative and quantitative data: - Chitwan Valley Family Survey (Ghimire et al. 2017)^79^ - Standardized Precipitation and Evapotranspiration Index (Vicente- Serrano et al. 2010)^78^

## Results

**Figure 1:**
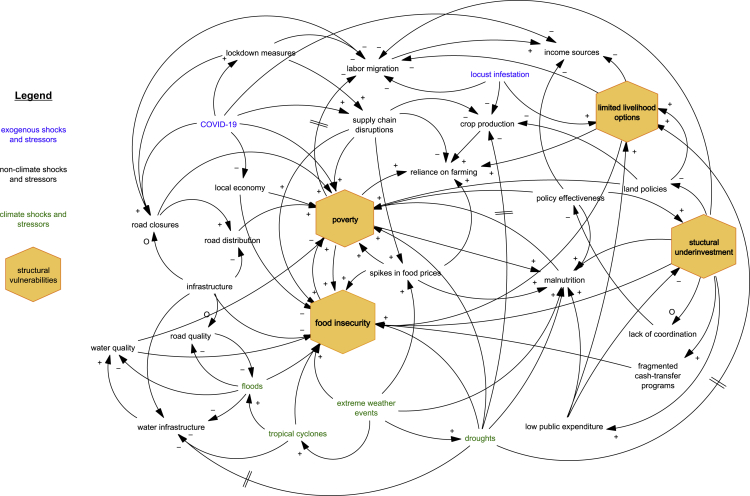
Vulnerability pathway model showing how structural vulnerabilities interact with labor migration in Madagascar ‘+’ symbolizes that the direction of the impact in a resulting variable has an increasing impact, while a ‘-’ symbolizes a decreasing impact. ‘O’ indicates a reversing link. The double lines indicate delays.

We find substantial differentials between different types of pre-existing vulnerabilities and compound events (CEs) on migration outcomes, with different ramifications for adaptive capacity across geographic and temporal contexts (see Table 1 for an overview). These results highlight that hazard and exposure are not the sole drivers of mobility outcomes resulting from compound drought impacts; rather, a combination of socio-economic pre-conditions and drought events also drive of these outcomes. During the 2021 drought in the Grand Sud of Madagascar, structural vulnerabilities (lack of road infrastructure) and systemic risk (the COVID-19 pandemic) limited the ability of households to migrate in response to drought risks (Figure 1). In other cases (e.g., Mexico and Nepal), structural vulnerabilities (lack of irrigation access) and/or CEs (e.g., consecutive droughts) increased the sensitivity of internal migration to drought risks.

### Vulnerability components in Madagascar

The well below average 2019/2020 rainy season in southern Madagascar (Le Grand Sud) resulted in crop losses, water- and food insecurity.^42^ Climate change was suspected to be a leading cause of food insecurity.^43^ However, Harrington et al.^23^show that factors other than drought attributable to climate change are the main drivers of food insecurity in Le Grand Sud. Mapping these lines of qualitative evidence in a VPM, we find that drought is a chronic risk in Le Grand Sud which affects multiple sectors. Road and water infrastructure are particularly poor in this region. During the rainy season, the roads can become impassable leading to infrastructure cut-offs from the rest of the country.^44^^,^^45^ In turn, access to markets and market-related internal labor migration is impinged. Resulting increases in food prices and barriers to humanitarian responses such as food aid show the interaction between structural vulnerabilities and food insecurity outcomes in the absence of drought. In this connection, the COVID-19 pandemic is an important risk multiplier affecting human mobility and food security outcomes during the drought event.^46^COVID-19 related lockdown measures reduced livelihood opportunities, including declining wages,^47^^,^^48^ resulting in restricted options for mobility. After the lifting of pandemic restrictions, labor-related mobility was impeded through road closures in cities that remained under lockdown.^49^ The VPM also shows how the measures to contain the virus limited human mobility for labor reasons or for getting out of harm’s way during the drought. Lockdown measures reduce people’s ability to pursue migration as an income source and contribute to supply chain disruptions. Before COVID-19, the interplay of poverty, structural underinvestment in development initiatives, and land use policies that have already diminished migration options for pastoralists. The main interactions between food insecurity and extreme weather events serve as one example of compounding factors that ultimately affect food insecurity outcomes. At the same time, the impact of drought on malnutrition is also exacerbated by structural underinvestment and contributes to poverty, thereby restricting migration options.

### Impacts from compound droughts on mobility outcomes in Mexico

We consider both temporally (such as a succession of drought in each community) and spatially compounding drought events in communities with different structural levels of structural vulnerabilities (such as absence of irrigation). We estimate their impact on internal migration decisions.

Overall, our results show that droughts increase the likelihood of internal migration in Mexico; the positive effects of droughts on migration are strongest in middle-wealth households (not the poorest, who likely experience credit constraints to migration) and in communities without irrigation. We also find evidence of compounding, where dry weather in the more distant past (t-2) and the more recent period (t-1) both create a migration response, but only in low-irrigation communities. Similarly, we observe spatial spillover effects, where dry weather in neighboring communities creates a migration response that is similar in magnitude to one emerging from experiencing dry weather within the community boundaries, but again, only in low-irrigation regions (see also Table S3). These results suggest that structural factors, such as a presence of irrigation systems, interact with drought events in creating a migration response. The results do not support, however, temporal compounding (where drought in (t-2) would moderate the impact of drought in (t-1)) or spatial compounding (where drought in a nearby community would dampen or amplify the impact of drought within the index community) for high irrigation communities (see also Table S2).

In the baseline model (Table 2), dry weather during the prior corn season (relative to a community’s own average baseline in 1980-1990) is associated with a 24 basis points increase in the probability of internal migration. This positive effect remains robust to the inclusion of individual, household, and community indicators. The negative effect of hot weather, by contrast, is no longer statistically significant after we adjust for individual (age, sex, whether person is the household head, years of education), household (properties, labor force in agriculture, prior internal and international migrants), and community (share of internal and international migrants, share in agriculture, presence of irrigation) indicators. Probability of internal migration declines with age; it is higher for men and household heads; and it increases with years of education. Households with either no or some agricultural involvements are more likely to send migrants relative to households that are fully invested in farming. Internal migration is more likely from households with prior internal migrants and less likely from households with prior US migrants. Internal migration is also more likely from communities that are more involved in agriculture (measured by the share of men working in agriculture), and those that have medium or high shares of internal migrants. Prevalence of US migration in the community or the presence of irrigation are not significantly associated with internal migration.

**Table 2:** Coefficient estimates from linear probability models of first internal migration in 93 agricultural communities in the Mexican Migration Project

Variables	(1)	(2)
Precipitation (ref: normal)		
Dry in (t-1)	0.0024(0.0008)∗∗∗	0.0014(0.0006)∗∗
Wet in (t-1)	-0.0006(0.0008)	-0.0003(0.0006)
Temperature (ref: normal)		
Hot in (t-1)	-0.0013(0.0006)∗∗	-0.0007(0.0005)
Cool in (t-1)	0.0002(0.0009)	0.0006(0.0006)
Age		-0.0003(0.0000)∗∗∗
Sex (0: female, 1: male)		0.0021(0.0004)∗∗∗
Household head? (0/1)		0.0029(0.0005)∗∗∗
Years of education		0.0004(0.0001)∗∗∗
Household properties (ref: none)		
Medium		0.0011(0.0008)
High		-0.0008(0.0010)
Household labor force in agriculture (ref: all)		
Some		0.0015(0.0005)∗∗∗
None		0.002(0.0004)∗∗∗
Household has prior internal migrants (0/1)		0.0056(0.0005)∗∗∗
Household has prior US migrants (0/1)		-0.0021(0.0005)∗∗∗
Community share of men in agriculture		0.0062(0.0023)∗∗∗
Community share ever migrated internally (ref: low)		
Medium		0.0023(0.0007)∗∗∗
High		0.0066(0.0008)∗∗∗
Community share ever migrated to US		0.0008(0.0020)
Community has no irrigation (0/1)		0.0032(0.0024)
State x year fixed effects	yes	yes
N (person-years)	436,978	436,978
R^2^	0.004	0.010

∗p<0.1, ∗∗p<0.05, ∗∗∗p<0.01. Standard errors (corrected for clustering at the community level) are in parentheses. Precipitation (temperature) deviation equals rainfall (maximum number of consecutive days over 30°C) in a community during corn season last year minus the mean value in community in 1980-1990, divided by standard deviation in that period. A community-year is wet (dry) if rainfall is one standard deviation or higher (lower) than its baseline mean, and normal otherwise. Temperature deviation categories are computed similarly. Corn season is June–February in Yucatan; September–March in Baja California, Chihuahua, Nayarit, Sinaloa and Sonora; and May–December in other states.

**Figure 2:**
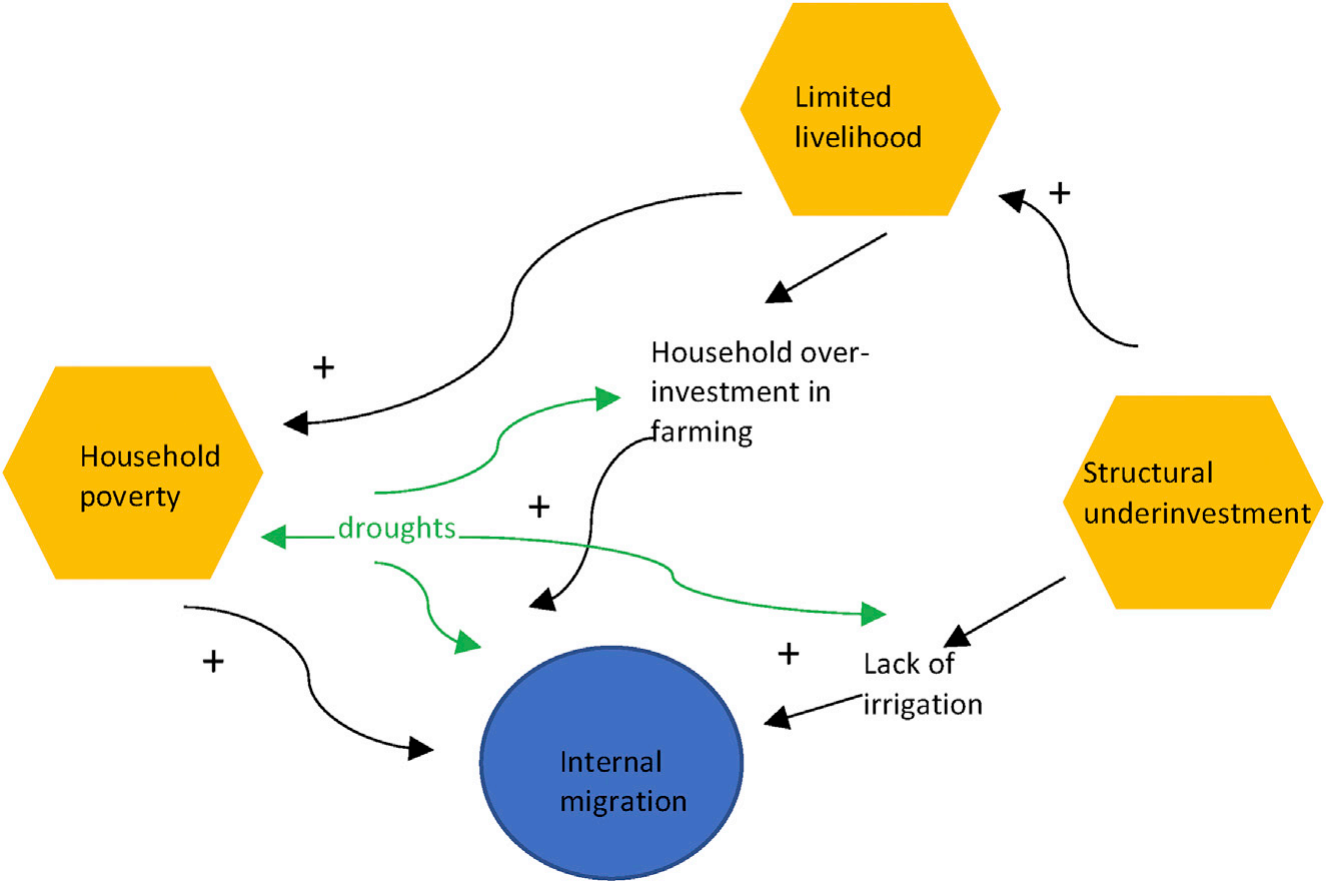
VPM of structural vulnerabilities (orange), drought impacts and internal migration in Mexico

We are also interested in the heterogeneity in the drought-migration link by structural vulnerabilities (Table 3). We consider two potential resources for vulnerabilities that likely moderate the impact of dry weather shocks: (1) household wealth and (2) community irrigation. We find significant variation in weather-driven migration responses across both dimensions. First, the positive impact of dry weather on migration is highest in medium-wealth households (model 1). This result confirms prior work on selectivity of migrants in Mexico, where poorer migrants often lack the resources to finance a move, and the wealthier migrants lack the desire to do so.^50^ Second, the positive effect of dry weather on migration is higher in communities with no irrigation, hence no buffer to overcome dry-weather shocks to farming (model 2). Of interest, neither household wealth nor community irrigation have a direct effect on the probability of migration, but each seems to exert an indirect effect through dry-weather shocks (Figure 2).

**Table 3:** Coefficient estimates from linear probability models of first internal migration in 93 agricultural communities in the Mexican Migration Project testing heterogeneity of precipitation effects

Variables	Precipitation effects by household wealth	Precipitation effects by community irrigation
	(1)	(2)
Precipitation (ref: normal)		
Dry in (t-1)	0.0010(0.0006)∗	0.0012(0.0006)∗
Household properties (ref: none)		
Medium	0.0006(0.0007)	0.0011(0.0008)
High	-0.0013(0.0010)	-0.0008(0.0010)
Precipitation x Properties		
Dry in (t-1) x Medium	0.0048(0.0024)∗	
Dry in (t-1) x High	0.0049(0.0040)	
Community has no irrigation (0/1)	0.0032(0.0024)	0.0029(0.0023)
Precipitation x No community irrigation		0.0065(0.0027)∗∗
Weather indicators	yes	yes
Controls	yes	yes
State x year fixed effects	yes	yes
N (person-years)	436,978	436,978
R^2^	0.010	0.010

∗p<0.1, ∗∗p<0.05, ∗∗∗p<0.01. Standard errors (corrected for clustering at the community level) are in parentheses. Precipitation (temperature) deviation equals rainfall (maximum number of consecutive days over 30°C) in a community during corn season last year minus the mean value in community in 1980-1990, divided by standard deviation in that period. A community-year is wet (dry) if rainfall is one standard deviation or higher (lower) than its baseline mean, and normal otherwise. Temperature deviation categories are computed similarly. Corn season is June–February in Yucatan; September–March in Baja California, Chihuahua, Nayarit, Sinaloa and Sonora; and May–December in other states. All models control for weather (dry, wet, hot, cool), individual (age, sex, whether person in household head, years of education), household (former internal and US migrants), community characteristics (share in agriculture, share with internal and US migration experience) and state-by-year fixed effects.

As we are particularly interested in compound impacts, we test for the temporal compounding of dry-weather shocks (see also Table S2). The findings suggest no temporal compounding of droughts effects on migration in Mexico.

In communities where less than a fourth of the cultivated land is irrigated, dry weather in the previous one to two years is associated with an increased probability of internal migration. The estimated effects for both years are roughly equal in size. In communities with a fourth or more of the land irrigated, by contrast, dry weather in either period does not lead to changes in internal migration. This finding suggests that structural resources (such as irrigation) can mitigate compounding of weather effects on migration. Similarly, when considering the spatial spillover of precipitation effects (see also supplemental information SI B, Table S1), we find that structural resources or vulnerabilities also play a key role in compounding weather effects on migration across space.

### Impacts from future compound droughts on mobility outcomes in Nepal

**Figure 3:**
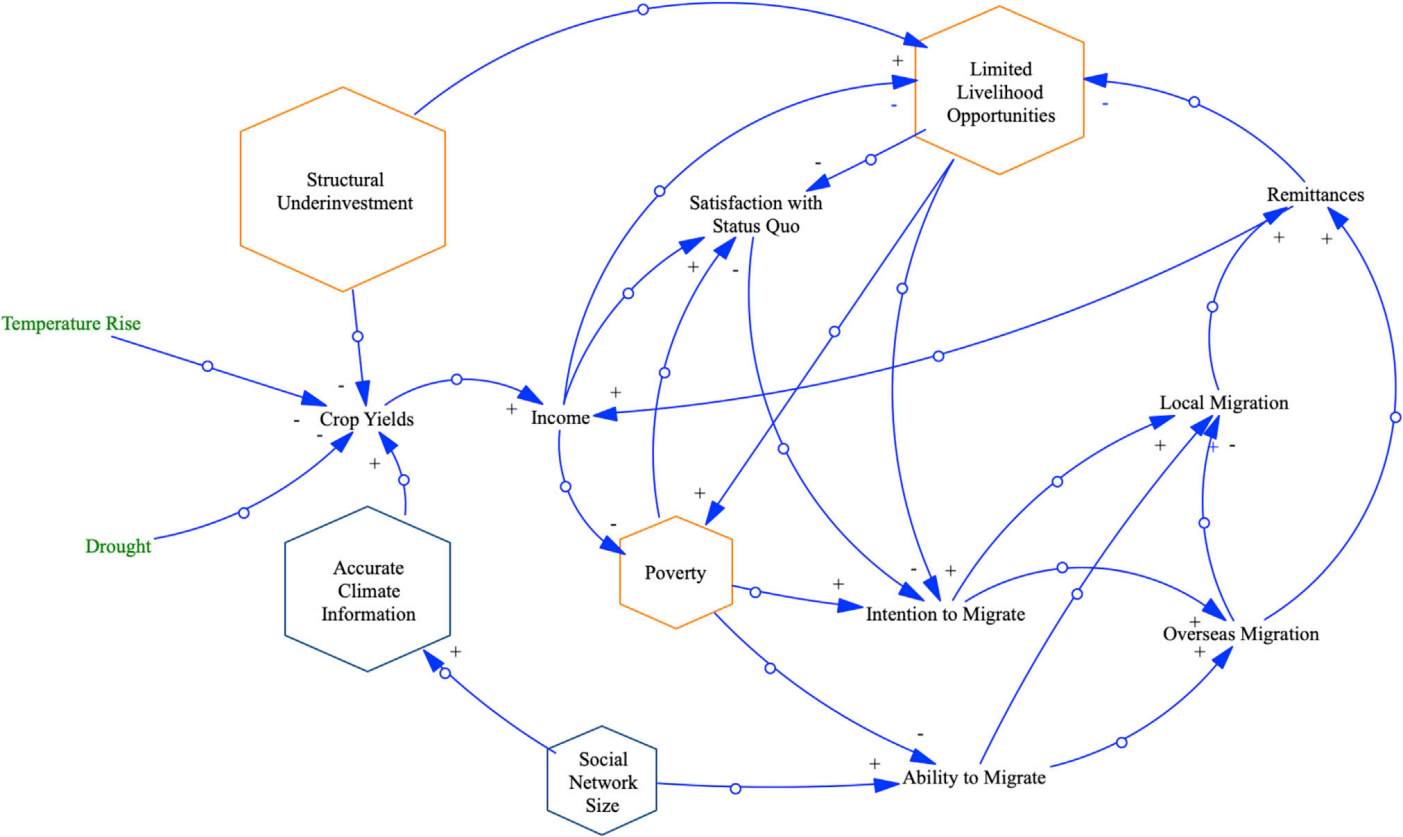
Causal loop diagram of structural vulnerabilities and migration drivers in Nepal Orange hexagons represent structural vulnerabilities also applicable in other case studies in this paper. Blue hexagons represent specific structural vulnerabilities that are assessed in this case study.

Here, we assess how compound drought and connected socio-economic pre-conditions may affect future mobility patterns from the Chitwan District in south-central Nepal, a region in which the majority of households engage in subsistence agriculture,^51^ with a combination of a VPM and an ABM (see also supplemental information SI C for details). We first construct a simplified VPM (Figure 3) based on factors affecting the use of rural livelihood portfolio options, including the use of rural-urban migration.^52^ These factors include individual household-scale variables, such as access to accurate climate information^53^^,^^54^; community-scale variables, such as the size and composition of one’s social network^55^^,^^56^; and macro-scale variables, e.g., investment in economic opportunities^52^^,^^57^^,^^58^ and climatic changes affecting crop yields.^59^^,^^60^^,^^61^The simplified VPM highlights how macroscale factors e.g., climate change and structural underinvestment may be mediated by meso- and household-scale factors. For example, access to larger social networks and more accurate climate information may attenuate climatic impacts on crop yields by facilitating the adoption of drought-resistant crop varieties. Migration may provide a negative feedback loop that helps to counter poverty traps, in part through remittances that bolster household income – but only if the ability to migrate is not critically affected by factors e.g., social isolation that may reduce the availability of these options. These relationships are used to create the scenarios of CEs, and drought with social pre-conditions (Table 4), that are formally analyzed via the ABM.

**Table 4:** Summary of ABM experimental scenarios

Scenario	Long-term climate trends	Social networks	Climate information	Drought year
1) Base Case	Stationary	Scale-free with avg degree = 4.5	Partial access	None
2) Single Drought	Stationary	Scale-free with avg degree = 4.5	Partial access	Drought in year 2025
3) Consecutive droughts	Stationary	Scale-free with avg degree = 4.5	Partial access	Drought in years 2024-2025
4) Drought + Previous temperature spike	1.5 °C increase in temperature in year before drought	Scale-free with avg degree = 4.5	Partial access	Drought in year 2025
5) Drought + No Info	Gradual 1.5 °C increase in temperatures by 2050	Scale-free with avg degree = 4.5	No access	Drought in year 2025
6) Drought + Full Info	Gradual 1.5 °C increase in temperatures by 2050	Scale-free with avg degree = 4.5	Full access	Drought in year 2025
7) Drought + Social Isolation	Gradual 1.5 °C increase in temperatures by 2050	Uniform with avg degree = 1	Partial access	Drought in year 2025
8) Drought + Full Network	Gradual 1.5 °C increase in temperatures by 2050	Uniform with avg degree = 99 (i.e. the number of other households in a community)	Partial access	Drought in year 2025

**Figure 4:**
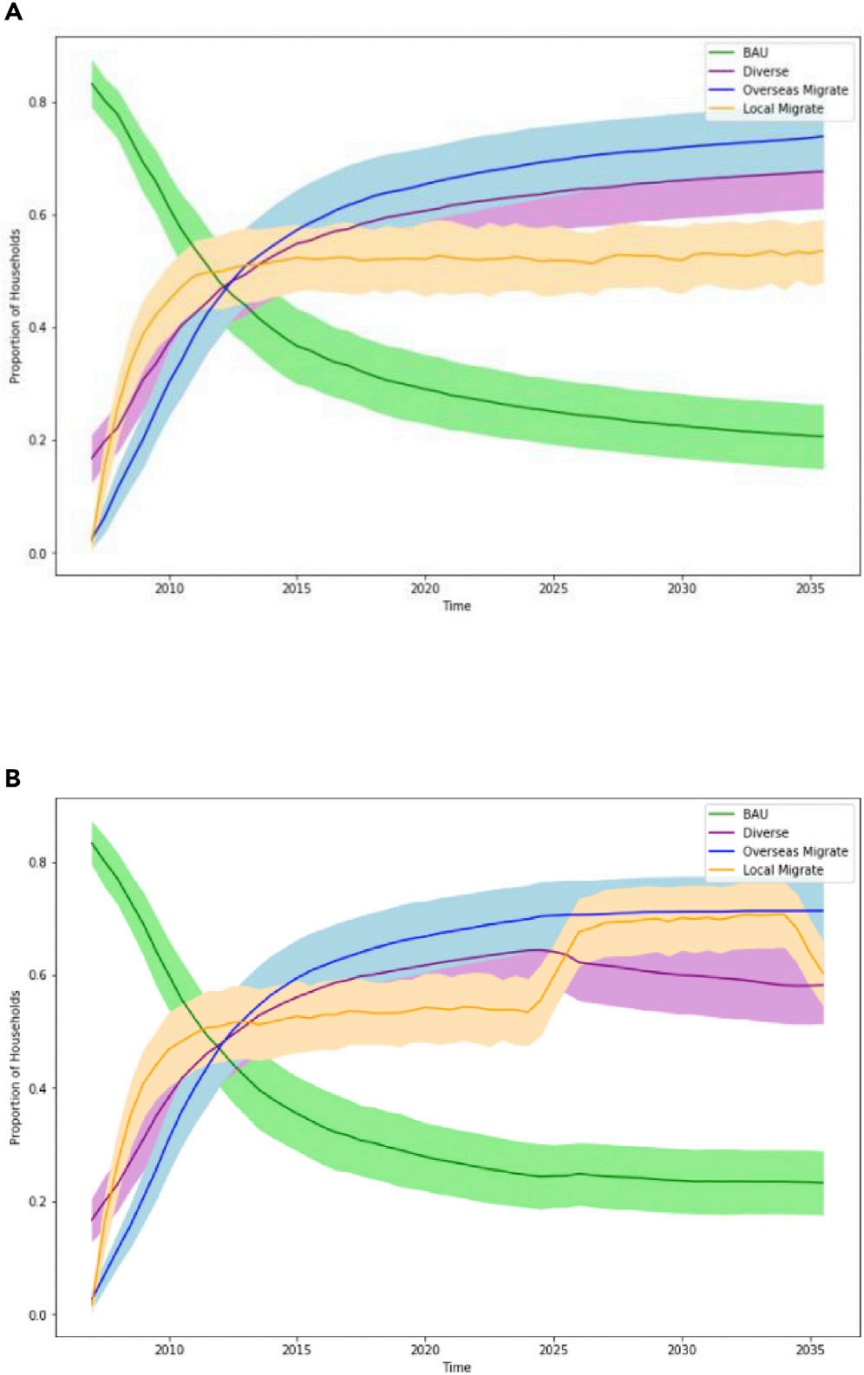
Household Livelihood Strategies Lines indicate the proportion of households opting for business-as-usual subsistence crops (green), diversified cash crops (purple), sending at least one migrant to a local destination (orange), and sending at least one migrant to an international destination (blue) over the simulated timeframe (2007–2035). These are shown for (1) Base Case conditions without shocks and under a stationary climate (panel A), and for (3) Consecutive Droughts in 2024–2025 (panel B). Shaded regions indicate +/− 1 SD over 1000 model simulations. Note that proportions may sum to more than 1, as households may pursue multiple types of migration and a farming strategy simultaneously.

Under (Condition 1) Base Case conditions (i.e., no shocks and a stationary climate), local migration rates are expected to stabilize by 2025–2026, such that there is no net change in households pursuing local migration during this time (orange line, Figure 4A). By contrast, overseas migration continues to increase slightly during this time (blue line, Figure 4A) because of the effect of emerging migrant networks that continue to lower the high cost of international migration for other households in the community.

We find that (Condition 3) a compound event in the form of two consecutive drought years (2024–2025) significantly changes these migration patterns. In the year following the droughts, an additional 13.5% of households engage in local migration (orange line, Figure 4B). This change represents both *ex-post* coping strategies and *ex ante* adaptation strategies among farming households. In the former case, more households engage in local migration to meet their basic needs after an extreme event. In the latter case, the consecutive droughts lower households’ expectations of future farming incomes, and more households migrate proactively to optimize their incomes across a portfolio of livelihoods. On the other hand, the occurrence of consecutive droughts slows the rate of overseas migration (Figure 4B, blue line): only an additional 0.49% of households take up this higher-cost migration in the post-drought year, compared to 1.26% during the same period in the Base Case. This illustrates a small but significant credit constraint imposed by the consecutive droughts; some households that otherwise would have sent an overseas migrant during this period can no longer afford to after the consecutive droughts.

**Figure 5:**
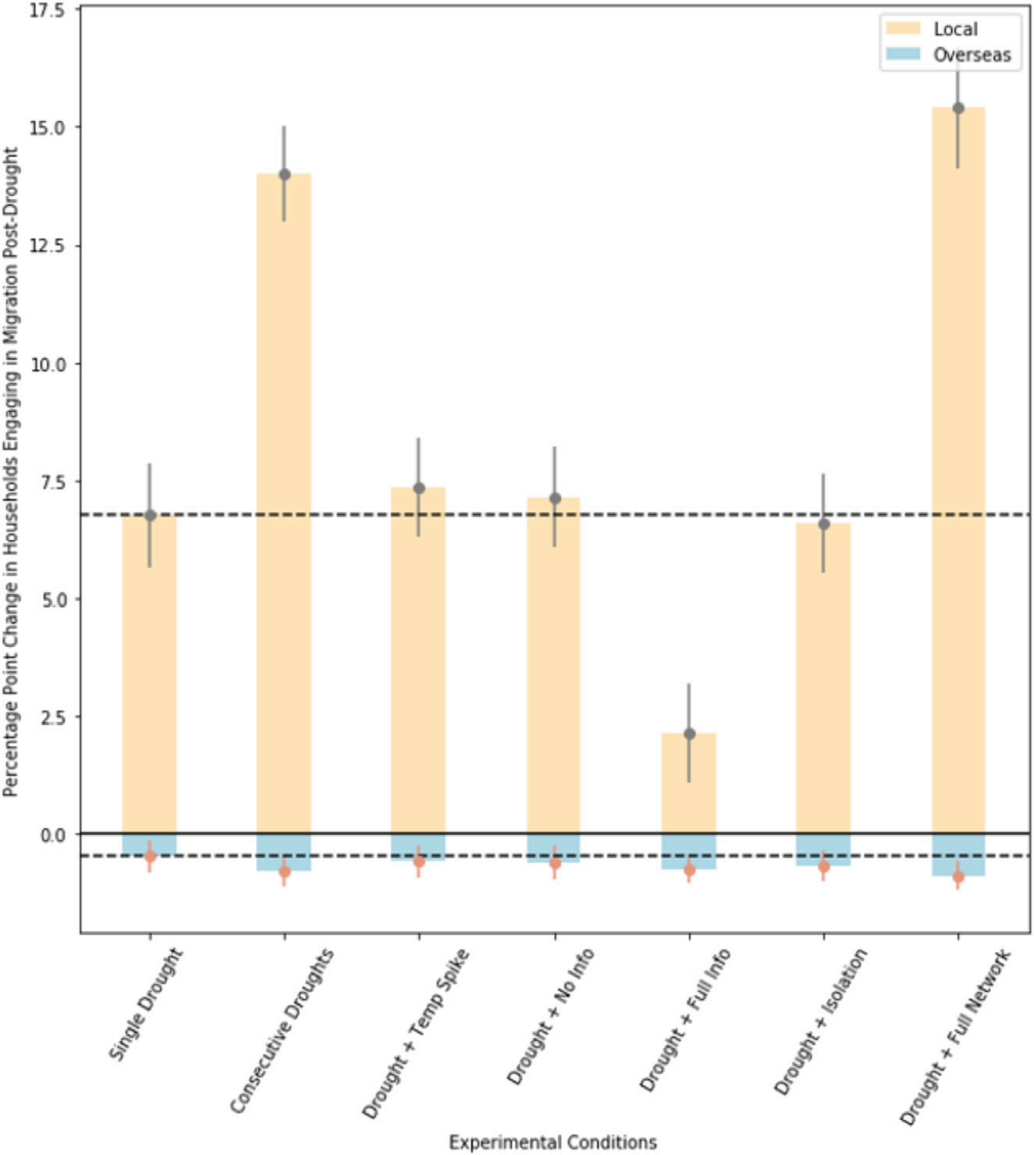
Net percentage point change in households engaging in local (orange) and overseas (blue) migration, relative to Base Case conditions, for seven forms of compound or connected events Error bars display the 95% confidence interval over 1000 simulations for each combination of condition/migration destination, and dotted lines illustrate the average change in local and overseas migration for the Single Drought condition.

We summarize these results for all experimental conditions in Figure 5, where we display the net percentage point change in households engaging in local migration (orange) and overseas migration (blue) across all seven experimental conditions, relative to Base Case conditions. Each experimental condition significantly increases migration to local destinations while decreasing migration to overseas destinations, relative to the Base Case. For example, a single drought year in 2025 increases the proportion of households engaging in local migration the following year by 6.8% points, while decreasing those engaging in overseas migration by 0.48% points relative to the Base Case. Across all conditions, drought increases household perceptions of risks to farming livelihoods, increasing the desire to engage in migration as an income diversification strategy. At the same time, the occurrence of drought reduces most household incomes, thereby diminishing the capacity to engage in high-cost overseas migration. Therefore, the main effect of drought is to direct more households to send a migrant to local destinations as a short-term coping strategy.

Compared to (Condition 2) a single drought without compound events or pre-conditions, three of the experimental conditions lead to significantly different migration patterns. Both Consecutive Droughts (3) and the Drought + Full Network (8) conditions significantly increase local migration relative to the Single Drought (2) condition. For the Consecutive Droughts condition, the compounding effect of multiple droughts on household incomes motivates a greater number of households to re-evaluate their *status quo* livelihood strategies and leads them to perceive greater risks to farming incomes. Although the droughts prevent most households from accumulating sufficient resources to engage in costly international migration, local migration is a feasible risk diversification strategy for most. The Drought + Full Network condition operates through a different mechanism: large social networks reduce costs for prospective migrants, enabling more households to engage in this livelihood once they perceive an increased threat to farm incomes. Perhaps counterintuitively, this precondition also leads to a significant decrease in the households engaging in overseas migration after a drought. However, this may be because larger networks accelerate the overseas migration process before the drought. Households that have not engaged in such migration by the time of the drought tend to be among the poorer members of the community and are unable to employ this strategy even when connected to large networks.

Conversely, the Drought + Full Info condition (6) significantly attenuates the spike in local migration. With the precondition of access to accurate information about climate and livelihood options, households are better able to proactively make livelihood decisions based on expected risks to their livelihoods before the occurrence of a shock. Therefore, a single drought under these conditions is less likely to significantly alter migration patterns compared to a drought without good access to accurate information.

### Synthesis

The multi-method, multi-location research design elucidated several compounding interactions and mobility outcomes across the three case studies. The vulnerability pathways identified can be interlinked well with quantitative results to determine human mobility outcomes (Table 5).

**Table 5:** Key results of the multi-method research across the three study locations

Study location	Compound Interaction	Effect on Mobility Outcomes
Madagascar	Drought vulnerability + COVID-19	Increased immobility
Mexico	Temporally compounding droughts	Increased mobility (low- irrigation); No discernible effect (high- irrigation)
Nepal	Temporally compounding droughts Drought + full social networks	Increased internal migration Decreased international migration

## Discussion and conclusion

Carefully disentangling vulnerability components that drive outcomes of compound drought events, we find that compound events (CEs) may significantly impact the mobility responses of vulnerable households to drought. However, there are substantial differences in the direction and size of this effect. In some cases (e.g., low-irrigation Mexican communities and internal Nepali migration), CEs may increase migration flows, whereas in others (e.g., Madagascar and international Nepali migration), such events may attenuate migration flows. These seemingly disparate effects on mobility highlight the importance of considering the many ways in which events and pre-conditions can compound with climate hazards in different contexts. Our three main findings are as follows: First, structural vulnerability is a main driver of compound drought impacts in Madagascar, Mexico, and Nepal. Our estimates suggest that pre-existing vulnerabilities, namely structural and socio-economic vulnerability components, exacerbate compound drought impacts on subsistence populations. In some cases (e.g., Madagascar), they attenuate mobility responses, whereas in other cases (e.g., Mexico and Nepal), they may increase internal migration flows. This agrees with the findings of Otto et al.,^24^ who found that back-to-back storms in Madagascar, Mozambique, and Malawi, together with pre-existing social vulnerability factors, left populations without time to recover, and together with climate change made the impacts of recent cyclones severe. Indeed, several articles in the climate change impact literature focus on heterogeneous effects on vulnerable populations, showing that migrants are often disproportionately exposed to (or affected by) drought risk.^20^^,^^21^^,^^62^ The evidence presented here also confirms and extends arguments for investigating how impacts from CEs drive vulnerability factors relevant to climate mobilities.^63^^,^^64^^,^^65^ However, our results extend findings from previous studies, which predominantly evaluate single drought occurrences and impacts on human mobility, showing migration is most common in dry land rural areas of low-income countries.^33^^,^^66^^,^^67^^,^^68^

Second, we find that there is a threshold in migration as an adaptation option to compound drought. In Nepal, we find that populations run out of resources to pursue overseas migration, which presents limitations for climate-resilient development options. This finding is in agreement with a large body of literature suggests migration as an adaptation mechanism in response to extreme weather events when other forms of adaptation are insufficient.^17^^,^^29^^,^^30^^,^^69^ Many studies suggest that climate hazards are a risk factor that may result in multiple disruptions of voluntary migration, with displacement situations within countries occurring.^70^^,^^71^^,^^72^^,^^73^

Our findings speak to several studies that have explored adaptive capacities of populations vulnerable to drought.^20^^,^^22^^,^^74^ These studies suggest that socio-economic differences shape populations’ ability to absorb drought impacts. However, these studies are typically based on a single method analysis and/or a single setting and fall short on providing systematic insights on CEs.

Third, our study setting highlights nuances in how individual components of drought vulnerability (i.e., hazard, exposure, sensitivity, and adaptive capacity) may be conceptualized through different methods. Our multi-method, multi-location approach is complementary and allows us to carve out various interacting factors of vulnerability on climate mobilities in the context of compound drought events. The usage of the VPM provides an overview on how vulnerability and risk factors compound. These pathways were then tested and quantified in an LPM in Mexico and an ABM in Nepal compound drought settings. The LPM provides a method to infer interactions between compound droughts and/or pre-conditions from historical mobility data, whereas the ABM offers a tool to prospectively identify compound effects through the creation of counterfactual scenarios. Whereas this multi-method approach allows us to tease out nuanced interactions between risk and vulnerability, it also bears several limitations. The VPM provides a static high-level overview of vulnerabilities components but does not show a dynamic or incremental change over time. ABMs typically rely on assumptions about underlying causal mechanisms that may be difficult to validate in the absence of other methods. LPMs estimated on observational data often suffer from sample selection and endogeneity issues. We attempt to circumvent these issues through a combined use of these individual methods.

The findings are not just relevant for expanding the empirical evidence on compound event impacts, but also for improving the design of policy interventions. In Madagascar, Mexico and Nepal, an awareness of compound event risks and the identification of potential impact areas can feed into anticipatory humanitarian action, preparing, and supporting the places and vulnerable populations likely to be impacted by CEs. This includes early attention to populations at risk of involuntary immobility.^18^ Our results underpin the implementation of drought plans with a focus on internal migrants, together with effective and well-targeted communication interventions to improve climate resilience development. These should aim at building awareness for the efficacy of migration as adaptation measures to CEs. In the Madagascar case, recent studies highlight the need to incorporate vulnerability and exposure components in extreme event attribution. In the Nepal context, and potentially other regions that are still developing climate information services, the findings represent a potential intervention for policymakers seeking to promote more orderly migration responses to changing environmental conditions: access to accurate information about livelihoods and climate forecasts can attenuate migration spikes that we might otherwise expect in their absence. Similarly, in the Mexican context, investing in irrigation systems in communities facing extreme weather risks can give individuals options to adapt in place rather than leaving them with no other option but to migrate to another region for work. A systemic approach to evaluating compound drought risks can assist policymakers in building adaptive capacity among their populations.

## STAR★Methods

### Key resources table

**Table:** 

REAGENT or RESOURCE	SOURCE	IDENTIFIER
**Deposited data**		

Migration data for Mexican case study	Mexican Migration Project	https://mmp.opr.princeton.edu/
Daily weather data for Mexican case study	NASA Earth Observing System Data and Information System	https://daac.ornl.gov/cgi-bin/dsviewer.pl?ds_id=1328
Migration and cropping data for Nepal case study	Agriculture and Migration Survey Calendar Data (Public Use), Chitwan Valley Family Study	https://cvfs.isr.umich.edu/data/data-documentation/household-level-data/
Drought data for Nepal case study	Standardized Precipitation and Evapotranspiration Index	https://spei.csic.es/database.html
Linear Probability Model for Mexican Case Study	This paper	Public code available at GitHub: https://github.com/fgarip/compoundevents (Aggregation of climate data requires approval for restricted-use geodata from MMP)
Agent-Based Model for Nepal Case Study	This paper	ZENODO: https://doi.org/10.5281/zenodo.7212485

**Software and algorithms**		

VenSimv.8	Ventana Systems (Open-Source)	https://vensim.com/free-download/
Python v 3.8, Anaconda Distribution	Python Software Foundation (Open-Source)	https://www.python.org/downloads/

### Resource availability

#### Lead contact

Further information and requests for resources should be directed to the lead contact, L. Thalheimer (lt6322@princeton.edu).

#### Materials availability

This study did not generate new materials.

### Method details

#### Multi-method research design

We combine well-established methods in climate impact research: vulnerability pathway model (VPM), linear probability model estimated on survey data on migration choices (LPM) and an agent-based model (ABM). We provide further details on the model setups in the supplemental information SI A–C. Our multi-method, multi-location approach is conducted in an integrative, semi-sequential manner to reflect the compounding nature of drought impacts at various drought impact locales, timescales, and mobility types (see Table 5). The VPMestablishes correlations important for the LPM and the ABM and provides relevant context in terms of vulnerability components and interaction pathways to interpret human mobility outcomes. At the same time, the LPM provides causal evidence for the interaction pathways detected by the VPM. The LPM helps to identify spatial and temporal effects of droughts on mobility patterns, while the ABM tests interactions of droughts with compound heat waves and relevant social pre-conditions. As we focus on a multi-method application, we think we have covered the most relevant drought measures in our context.

#### VPM details

The VPM constructed for the Madagascar and Nepal case studies were designed using open-source software VenSimv.8.

#### Mexico case study details

Our main data come from the Mexican Migration Project (MMP)^75^ with retrospective life histories of 46,454 individuals between 1991 and 2018 (*N* = 436,978 person-years). These individuals reside in 93 rural communities (nested within 22 states) where at least 45 percent of the male labor force works in agriculture and where corn is often the dominant crop, see also Figure S1. The data include records of internal and international migration trips as well as information on individual characteristics, household demographic and economic composition, and community migration history and institutions over time. We merge the MMP surveys with daily gridded weather data obtained from the NASA Earth Observing System Data and Information System^76^ and based on the interpolation of information obtained from ground weather stations over 4 km^2^ grids (see also supplemental information SI B, section 2.2 and Figure S2).

Our baseline model connects weather deviations to internal migration decisions using a linear probability model:

$$y_{ijcst}=\beta^{\prime }w_{cst-1}+\delta^{\prime }x_{ijcst}+v^{\prime }h_{jcst-1}+\gamma^{\prime }z_{cst-1}+\alpha_{st}+\varepsilon_{ijcst}$$

where*y*_*ijcst*_ denotes the binary outcome of whether an individual *i* from household *j* in community *c* in state *s* took an internal migration trip for the first time in year *t*. The vector *w*_*cst-1*_ represents weather deviations for community *c* in year *t-1*. The vector *x*_*ijcst*_ includes individual characteristics (age, sex, whether person is a household head, years of education) that are likely to be related to migration choices. The vectorsβandδrepresent the coefficients for weather deviations and individual characteristics, respectively; prime (‘) denotes transpose. The vector *h*_*jcst-1*_ contains time-varying household attributes measured at *t-1.* The coefficient *v* represents any relationships between those attributes (household properties, share working in agriculture, whether the household has any former internal or international migrants) and internal migration. The vector *z*_*cst-1*_ denotes time-varying community characteristics (share ever migrated internally and internationally, whether community has irrigation) measured at *t-1* with the corresponding coefficient vector *γ*. The term α_*st*_ represents the fixed effects for state *s* at time *t*, and ϵ_*ijcst*_ is the error term. The fixed effects remove any time-specific shocks to states. The introduction of PROGRESA (a conditional cash transfer program that was reaching more than half of poor households by 2016) in 1997 might have reduced migration pressures in rural communities and its effects might be differentially distributed across states.^77^ The state-by-year fixed effects absorb such unobserved factors. Therefore, the resulting coefficients represent the average effects of indicators within units (individuals, households, communities) in a given state-year. We compute cluster-robust standard errors to account for any within-community correlation in migration decisions (see Table 2, see also Figure S1 and Table S3).

#### Nepal case study details

To model the effect of compound drought impacts on migration with future warming, we make use of the Standardized Precipitation and Evapotranspiration Index (SPEI)^78^ and survey data on migration from the Chitwan Valley Family Survey.^79^ We conduct a series of experiments using a previously developed and validated agent-based model (ABM) of how smallholder farmer livelihood choices, including migration, evolve in the context of changing climatic conditions,^80^ developed using open-source Python v. 3.8 software. Briefly, model agents represent farming households that can choose among a portfolio of livelihood strategies: farming low-risk, low-reward cereal crops; high-risk, high-reward cash crops; and/or sending one or more household members as a migrant to earn remittances (see also supplemental information SI 3.2 and Table S4 for details on the model construction and supplemental information SI 3.3 and Figures S3 and S4 for a model validation). For this latter option, households can further choose low-cost, low-reward local migration (e.g., to another destination in Nepal or India), or high-cost, high-reward overseas migration (e.g., to the Persian Gulf or Southeast Asia). In each time step representing a single cropping cycle (with an average of two cropping cycles per calendar year), households may decide to stick with their status quo portfolio of strategies or update their strategies if they are unsatisfied with their incomes, relative to their neighbors and their own recent history. In the latter case, households enter a boundedly rational decision-making process, in which they seek to maximize their expected income while minimizing income volatility, weighted by a risk aversion parameter. Households select the strategy portfolio that maximizes their utility, subject to budget and other constraints. While the ABM does not perfectly capture all trends related to farmer livelihood choices, it accurately predicts overall observed migration rates from 2006-2017. Qualitatively, the ABM also replicates important patterns related to the split between local and overseas migration, and the impact of extreme drought on migration rates, lending some confidence to the general conclusions that we draw below.

In all but one experimental condition, households have imperfect information, and rely on a combination of information from their social networks and government sources to form perceptions of livelihood costs and income distributions. Agents are heterogeneous in their risk preferences, access to government information, and starting wealth, and their livelihood risk perceptions are updated as they gather more information from their networks. Climate risks are incorporated in two ways: (1) an increase in average temperatures over time, which leads to a decrease in average crop yields^81^; and (2) stochastic extreme events, which are assumed to eliminate farming incomes in the cropping cycles in which they occur.

We first apply a VPM to assess how these climate events are likely to combine with structural vulnerabilities to affect both local and overseas migration outcomes (Figure 3). We then parameterize the ABM using data on farming costs and incomes in the Chitwan District from 2006-2015,^79^ as well as data on migration costs and remittances from Nepali migrants to India and other overseas regions, including the Persian Gulf, Southeast Asia, and East Asia,^82^ see Table 5. We construct a series of eight experimental scenarios: droughts in consecutive years, a compound event of a 1.5°C increase in mean annual temperature above normal followed by a drought, a drought with the precondition of no/full access to accurate climate information, and a shock with the precondition of social isolation/full social connection (see Table 4 for a summary of scenario descriptions). In each scenario, we run 1000 simulations of the model for the years 2007-2035. Shocks occur stochastically (with a 1-in-20-year probability) from 2007-2020, at which point we force the model with shocks based on the given scenario. For a single shock, we implement a drought in the two cropping cycles of 2025, and for consecutive shocks, we implement droughts in the four cropping cycles from 2024-2025. We then compare the proportion of households engaging in both local and overseas migration in the two cropping cycles immediately following the shocks with those rates in the two cropping cycles immediately preceding the shocks. We provide a synthesized results overview in Table 5 in the main text.

## Data Availability

• Data: This paper analyzes existing, publicly available data. These accession numbers for the datasets are listed in the key resources table. • Code: Original code related to the development of the ABM has been deposited at Zenodo and is publicly available as of the date of publication. The DOI is listed in the key resources table. Code for the VPM • Any additional information required to reanalyze the data reported in this paper is available from the lead contact upon request.
