# Supplementary material for: Compound impacts from droughts and structural vulnerability on human mobility
Source: iScience. 2022 Nov 23;25(12):105491. doi: 10.1016/j.isci.2022.105491 (PMC9801241; doi:10.1016/j.isci.2022.105491)
Supplement: Document S1. Figures S1–S4 [file mmc1.pdf]

iScience, Volume ■ ■

## Supplemental information

# Compound impacts from droughts

# and structural vulnerability on human mobility

**Lisa Thalheimer, Nicolas Choquette-Levy, and Filiz Garip**

Supplementary Information

**Compound impacts from droughts and structural vulnerability on human mobility**

# 1 Supplementary Information SI A

## 1.1 Brief country context Madagascar

Madagascar is one of the world's poorest countries.[1] Its population is affected by and exposed to recurring drought, cyclones and flood events that have compound and hit highly vulnerable communities.[2] The Internal Displacement Monitoring Center (IDMC) estimates that 197,565 people per year on average are displaced by sudden-onset hazards such as earthquakes, flood, storm surge and tsunamis. In 2020, an approximate 23,000 people were newly displaced as a result from climate and weather-related events. Contrary to other drought-prone areas, conflict is not a driving force of internal displacement in Madagascar.[3,4]

## 1.2 Vulnerability pathway model

We map evidence on vulnerability components in a vulnerability pathways model (VPM) to provide an overview on interlinkages of vulnerability and risk for the July 2019 to June 2021 drought (see Figure 1 in the main text). We first conducted a content review of the literature on vulnerability components. The VPM illustrates how structural vulnerability components (yellow) - poverty, structural underinvestment, and limited livelihood options - affect human mobility and drive food insecurity (red). We distinguish between exogenous non-climate shocks (blue), exogenous climate shocks and stressors (green) and endogenous non-climate shocks and stressors (black). Connections between VPM elements are drawn through arrows indicating an increasing (+) or decreasing (-) impact. We used the open-source software Vensim PLE, version 8 (see Key Resources Table for further details).

# 2 Supplementary Information SI B

## 2.1 Brief country context Mexico

Mexico is a developing country with over 40 percent of its population in poverty. 37 million Mexicans (36% of the population) live in rural areas where nearly 80% of agricultural production is rainfed. Corn is the most common crop. Rural populations are highly vulnerable to droughts, given the susceptibility of corn to drought damage and given low levels of crop diversification among farmers.[5,6]

Mexico has urbanized rapidly in the past fifty years; it has also supplied the largest sustained flow of international migrants in the world. Between 1960 and 2010, the share of the urban population has increased from 50 to 80%. In the same period, an estimated 12 million Mexicans have migrated to the United States.[7,8] Empirical findings suggest that gradual changes in weather, such as rainfall deficits or temperature extremes relative to a historical baseline, can instigate international migration as well as internal moves from rural Mexico.[9–11]

## 2.2 Data preparation

We aggregate weather data across grids in each community boundary and compute annual measures of precipitation and temperature. Given that most communities grow corn, we focus on planting-to-harvesting season for this crop. The season runs from June to February in Yucatan, from September to March in Baja

California, Chihuahua, Nayarit, Sinaloa, and Sonora, and from May to December in other states.[1] We consider two weather indicators (measured by total precipitation and maximum number of consecutive days over 30°C), extremes of which can be detrimental to corn production, leading to household budget constraints, and eventually to migration decisions. We express the weather indicators in deviation form. Precipitation (temperature) deviation equals rainfall (measured by maximum number of consecutive days over 30°C) in a community during the corn season in previous year minus the mean value in the community in 1980-1990, divided by standard deviation in the baseline period. A community-year is wet (dry) if rainfall is one standard deviation or higher (lower) than its baseline means, and normal otherwise. Temperature deviation categories are computed similarly. Table S1 provides descriptive statistics for all indicators used in analysis.

*Table S1: Descriptive statistics from 93 agricultural communities in the Mexican Migration Project data*

| Variable | | Mean | Standard deviation |
|---|---|---|---|
| First internal migration trip (0/1) | | 0.01 | 0.08 |
| Seasonal precipitation deviation | | | |
| | Dry | 0.11 | 0.32 |
| | Normal | 0.65 | 0.48 |
| | Wet | 0.24 | 0.43 |
| Seasonal temperature deviation | | | |
| | Hot | 0.24 | 0.42 |
| | Normal | 0.64 | 0.48 |
| | Cool | 0.13 | 0.33 |
| Age | | 34.26 | 14.34 |
| Sex (0: female, 1: male) | | 0.44 | 0.50 |
| Household head? (0/1) | | 0.48 | 0.50 |
| Years of education | | 7.02 | 4.14 |
| Household properties | | | |
| | None | 0.93 | 0.26 |
| | Medium | 0.04 | 0.20 |
| | High | 0.03 | 0.17 |
| Household labor force in agriculture | | | |
| | All | 0.23 | 0.42 |
| | Some | 0.30 | 0.46 |
| | None | 0.47 | 0.50 |
| Household has prior internal migrants (0/1) | | 0.24 | 0.42 |
| Household has prior U.S. migrants (0/1) | | 0.30 | 0.46 |
| Community share of men in agriculture | | 0.62 | 0.11 |

[1] This information comes from the Crop Calendar Dataset provided by the University of Wisconsin-Madison. https://sage.nelson.wisc.edu/data-and-models/datasets/crop-calendar-dataset/

| | | |
|---|---|---|
| Community share ever migrated internally | 0.15 | 0.11 |
| Low | 0.40 | 0.49 |
| Medium | 0.32 | 0.47 |
| High | 0.28 | 0.45 |
| Community share ever migrated to US | 0.14 | 0.12 |
| Community has no irrigation (0/1) | 0.05 | 0.22 |
| N (person-years) | 436,978 | |
| N (persons) | 46,454 | |
| N (households) | 12,900 | |
| N (communities) | 93 | |
| N (states) | 22 | |
| N (years) | 28 | |

Precipitation deviation equals seasonal rainfall (during planting-to-harvesting months for corn) in a community last year minus the mean value in community in 1980-1990, divided by standard deviation in that period. A community-year is wet (dry) if rainfall is one standard deviation or higher (lower) than its baseline means and normal otherwise. Temperature deviation (based on maximum number of consecutive days over 30C) is computed similarly.

### 2.3 Changes in migration probability over time

Our data cover a 19-year period (1991-2018). Propensities for internal migration might be changing in this period. Our main model (Table 1, column 2 in the main text) already includes state-by-year fixed effects. To better understand annual changes in internal migration probability, we changed this specification slightly and introduced state and year fixed effects (thus removing the interaction). Figure S1 shows the coefficient estimates (empty circles) and confidence intervals (thicker line for 95% and thinner one for 90%) for the binary indicators for each year between 1992-2018 (reference year: 1991) from this model. The estimates do not obtain statistical significance with two exceptions: the likelihood of migration is slightly higher in 2010 relative to 1991 ($p<0.05$), and it is slightly lower for 2018 ($p<0.05$).

*Figure S1: Coefficient estimates for binary year indicators (reference: 1991) from linear probability model of first internal migration in 93 agricultural communities in the Mexican Migration Project*

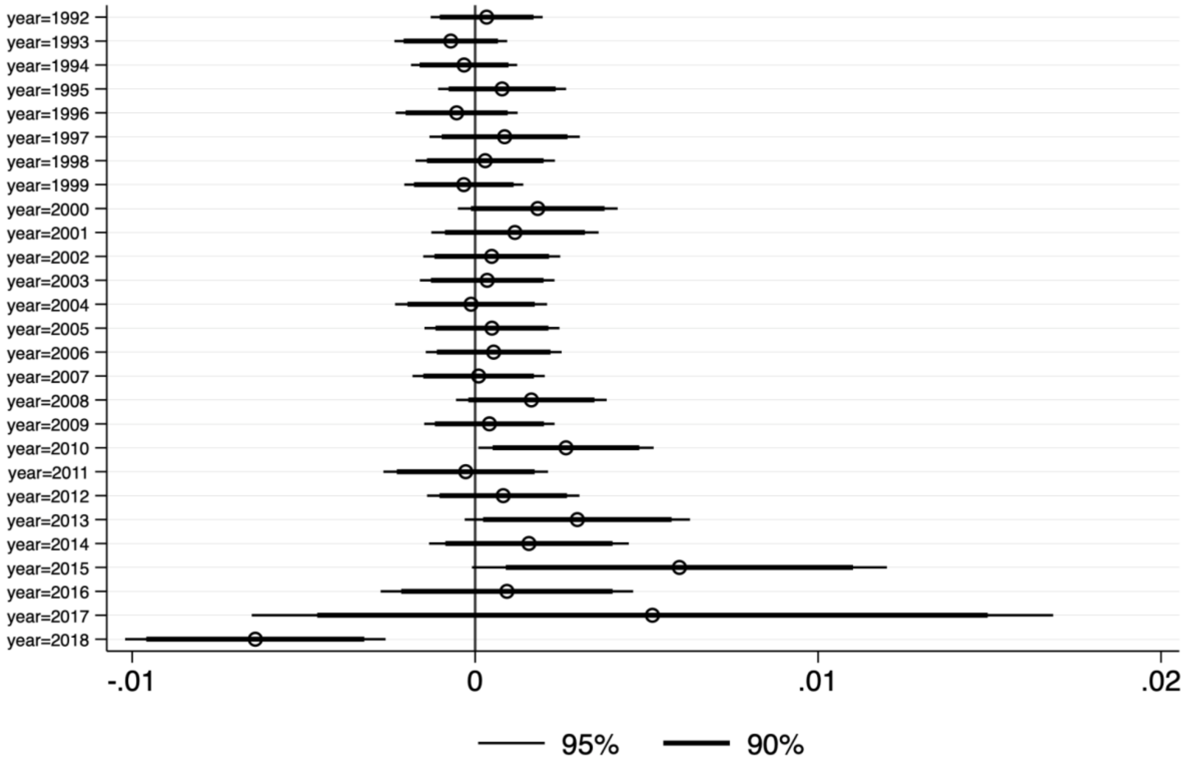


In an extended model, we introduced interactions between year indicators and experiencing dry weather. Figure S2 shows the coefficient estimates for the interaction terms. The results suggest that the effect of dry weather on migration probability showed some variation over time. In particular, exposure to dry weather had a relatively lower impact ($p<0.05$) on the likelihood of migration in 1993 compared to the reference year (1991), and a relatively higher impact ($p<0.05$) in 1997, 1998, 2008 and 2011. This pattern generally suggests that the effects of droughts might be intensifying over time, although it is not observed consistently in each consecutive year.

*Figure S2: Coefficient estimates for binary year indicators (reference: 1991) interacted with dry weather from linear probability model of first internal migration in 93 agricultural communities in the Mexican Migration Project*

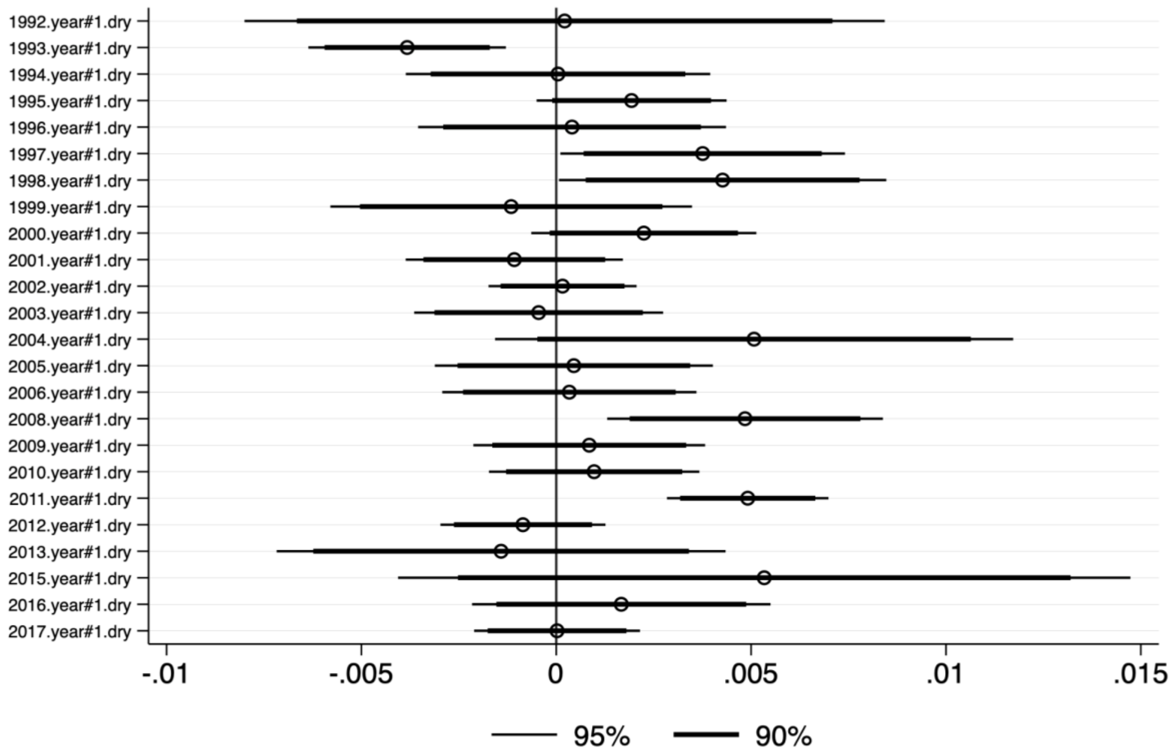


## 2.4 Temporal compounding

Model 1 includes indicators for dry weather in a previous year (t-1) and two years ago (t-2) as well as the interaction between the two. The results are indifferent to the baseline model (see Table 2 in the main text); only dry weather in the most recent period seems to matter to internal migration decisions (Table S 2).

*Table S2: Coefficient estimates from linear probability models of first internal migration in 93 agricultural communities in the Mexican Migration Project testing temporal compounding of precipitation effects*

| Variables | All communities | | Communities < 0.25% irrigated land | | Communities ≥25% irrigated land |
|---|---|---|---|---|---|
| | (1) | | (2) | | (3) |
| Precipitation (ref: normal) | | | | | |
| Dry in (t-1) | 0.0015 | ** | 0.0032 | ** | 0.0007 |
| | (0.0007) | | (0.0015) | | (0.0009) |
| Dry in (t-2) | 0.0004 | | 0.0034 | * | -0.0007 |
| | (0.0008) | | (0.0017) | | (0.0007) |
| Dry in (t-1) x Dry in (t-2) | -0.0007 | | -0.0020 | | 0.0020 |

| | | | |
|---|---|---|---|
| | (0.0016) | (0.0028) | (0.0019) |
| Weather indicators | yes | yes | yes |
| Controls | yes | yes | yes |
| State x year fixed effects | yes | yes | yes |
| N (person-years) | 436,978 | 175,590 | 261,388 |
| $R^2$ | 0.010 | 0.013 | 0.009 |

*p<0.1, **p<0.05, ***p<0.01. Standard errors (corrected for clustering at the community level) are in parentheses. Precipitation (temperature) deviation equals rainfall (maximum number of consecutive days over 30C) in a community during corn season last year minus the mean value in community in 1980-1990, divided by standard deviation in that period. A community-year is wet (dry) if rainfall is one standard deviation or higher (lower) than its baseline mean, and normal otherwise. Temperature deviation categories are computed similarly. Corn season is June-February in Yucatan; September-March in Baja California, Chihuahua, Nayarit, Sinaloa and Sonora; and May-December in other states. All models control for weather (dry, wet, hot, cool), individual (age, sex, whether person in household head, years of education), household (former internal and U.S. migrants), community characteristics (share in agriculture, share with internal and US migration experience) and state-by-year fixed effects.

## 2.5 Spillover effects

Next, we consider the spatial spillover of precipitation effects (Table S 3). Model 1 introduces a binary indicator for whether any other communities in an index community's state experienced dry weather shocks last year. The results in the pooled sample suggest no such spatial spillover of dry shocks. But, when we split our sample into two by community irrigation levels, we detect spillover effects from rainfall deficits in the state. Specifically, Model 2 shows that dry weather in neighboring communities increases the probability of migration at about the same rate as dry weather in one's own community in communities with little irrigation. Neither effect is significant in model 3 which is estimated on communities with a fourth of more of their land irrigated.

*Table S3: Coefficient estimates from linear probability models of first internal migration in 93 agricultural communities in the Mexican Migration Project testing spatial compounding of precipitation effects*

| Variables | All communities | | Communities < 0.25% irrigated land | | Communities ≥25% irrigated land |
|---|---|---|---|---|---|
| | (1) | | (2) | | (3) |
| Precipitation (ref: normal) | | | | | |
| Dry in (t-1) | 0.0014 | * | 0.0047 | *** | 0.0008 |
| | (0.0008) | | (0.0011) | | (0.0009) |
| Dry in other communities in state in (t-1) | 0.0001 | | 0.0043 | * | -0.0011 |
| | (0.0015) | | (0.0022) | | (0.0019) |
| Weather indicators | yes | | yes | | yes |
| Controls | yes | | yes | | yes |
| State x year fixed effects | yes | | yes | | yes |
| N (person-years) | 436,978 | | 436,978 | | 436,978 |
| $R^2$ | 0.010 | | 0.013 | | 0.009 |

*p<0.1, **p<0.05, ***p<0.01. Standard errors (corrected for clustering at the community level) are in parentheses. Precipitation (temperature) deviation equals rainfall (maximum number of consecutive days over 30C) in a community during corn season last year minus the mean value in community in 1980-1990, divided by standard deviation in that period. A community-year is wet (dry) if rainfall is one standard deviation or higher (lower) than its baseline mean, and normal otherwise. Temperature deviation categories are computed similarly. Corn season is June-February in Yucatan; September-March in Baja California, Chihuahua, Nayarit, Sinaloa and Sonora; and May-December in other states. All models control for weather (dry, wet, hot, cool), individual (age, sex, whether person in household head, years of education), household (former internal and U.S. migrants), community characteristics (share in agriculture, share with internal and US migration experience) and state-by-year fixed effects.

# 3 Supplementary Information SI C

## 3.1 Country context Nepal

Tucked between the Tibetan Plateau to the north and the Indo-Gangetic plain to the south, Nepal features a uniquely distinct set of ecological belts that shape its climate. The Himalaya Mountain range in the north, steep hills in the middle of the country, and fertile Terai plains in the south sit within an area approximately 250 km in distance from North to South. As such, Nepal is subject to a diverse set of climate impacts, including glacial melt and glacial lake outburst flooding; erosion and landslides in the mid-Hills region, and flooding and drought in the Terai.[12,13] While flooding and landslides have led to sudden displacement of populations in the Himalaya and mid-Hills, here we focus on the relationship between climate and mobility in the Terai, where 50 percent of the country's population resides.[14] Over 70 percent of the population relies on subsistence agriculture as a primary livelihood, and the small size of farms (average of 0.7 ha) and lack of access to credit leaves farmers highly vulnerable to climate-related shocks.[15,16] Out-migration from the region is already a common livelihood strategy, with more than 2.1 million Nepalis, or roughly 7.5 percent of the population, living abroad in 2021, and remittances from overseas migrants accounting for 24 percent of Nepal's GDP.[1,17]

## 3.2 Agent-Based Model Development

The agent-based model used for the analysis of migration from Nepal in section 3.3 of the main text is described in further detail in Choquette-Levy et al.[18] (see also STAR Methods). Two specific adjustments were made to facilitate a comparison to the Madagascar and Mexico case studies in this paper. First, the single migration livelihood option used in the study was disaggregated into two channels, "local" and "overseas", for this analysis (Table S4). Here, "local" migration is interpreted as outmigration from the Chitwan Valley to other destinations within Nepal or to India, and is characterized by low upfront costs, low expected remittances, and low variance in remittance incomes. "Overseas" migration is interpreted as outmigration to all other international destinations (e.g., Persian Gulf countries, Malaysia, Australia, etc.) and is characterized by high upfront costs, high expected remittances, and high variance in remittance incomes. For each channel, data for these three variables - cost, expected remittances, and variance in remittances - were taken from Shrestha[19], and reproduced below.

A second adjustment was to implement pre-specified climate forcings into the ABM, for ease of comparison between experimental scenarios. In Choquette-Levy et al.[18], a pre-specified rise in mean temperature occurs linearly over a period of 44 years (2007 - 2050), and droughts occurred probabilistically as a function of the mean annual temperature and its relationship to the distribution of a drought indicator. The same methods are applied in this study to model the impacts of mean temperature rise and probabilistic droughts from 2007-2020. Starting in 2020, we assume a stationary temperature and do not model droughts until the years 2024-2025. In these years, droughts and/or temperature rise are modeled deterministically based on the experimental conditions described in Table 4 in the main text. From 2026-2035, the model is run without any further droughts or temperature rise. This allows us to isolate the impact of the different experimental conditions in 2024-2025 on livelihood choices in subsequent years.

*Table S4: Parameterization of migration costs, expected incomes, and standard deviations for the "local" and "overseas" migration channels. Data are taken from Shrestha*[19].

| | Local Channel | Overseas Channel |
|---|---|---|
| Example Destinations | Nepal, India | Persian Gulf, Southeast Asia, East Asia |
| Upfront Migration Cost (USD/migrant) | 62.50 | 1130 |
| Mean Remittance Income (USD/migrant/cropping cycle) | 199 | 872 |
| Remittance Income Standard Deviation (USD/migrant/cropping cycle) | 188 | 703 |

### 3.3 Agent-Based Model Validation

While it is difficult for any ABM to perfectly replicate observed behavior of a complex system, a useful model should ideally be able to replicate patterns of behavior at different scales that are relevant to the proposed research questions.[20] Here, we validate the ABM used in this analysis against two observable patterns in the Chitwan Valley Family Study (CVFS) Labour Outmigration, Agricultural Productivity and Food Security survey[21] - the distribution of households by livelihood strategy and the number of migrants. We also compare ABM model predictions of aggregate migration behavior against two additional patterns: the relative proportions of migrants going to local vs. international destinations (taken from Nepal-wide data collected by Shrestha[19]), and the effect of extreme drought on annual migration rates. To conduct these validation tests, we calibrate the initial distribution of agents' livelihood strategies in the model using the first year of CVFS data (2006) and take the final year of the CVS survey data (2017) to validate model predictions. More precisely, we conduct 100 simulations of the model from 2006 through the first cropping cycle of 2017 and compare the distribution of predicted model outcomes to observed data from the survey for the year July 2016-July 2017. For the test involving the relative proportions of local vs. international migrants, we compare model predictions in 2017 to Nepal-wide data from Shrestha[19], also collected around the same time.

A comparison of the predicted distribution of agents by livelihood choice against observed data indicates that the model is relatively accurate in predicting the number of households engaging in migration, though it overestimates the number of households that adopt Cash Crops and substantially underestimates the households that continue with BAU crops (Figure S3). The CVFS data indicates that 71.4 percent of households had at least one migrant living outside the home in 2016-2017; on average, the model predicts 73.4 percent of households would engage in migration by this time. However, the CVFS data indicates that most farming households continue to pursue BAU crops by 2017 (64.4 percent), while the model predicts that an average of only 28.1 percent of households would have remained in this livelihood by the same year. Conversely, CVFS data indicates that 52.7 percent of households farmed some sort of cash crop in 2016-2017, while the average model prediction is 62.1 percent. This indicates that while the model may be capturing some of the underlying mechanisms motivating out-migration from the Chitwan Valley, it is missing some factors that lead to high persistence of BAU crops. Such factors may include government subsidies for basic cereal staples to ensure food security, specific perceived risks of switching to cash crops that are not captured by income distributions alone, and cultural preferences for certain cereals, e.g., rice and maize. Additionally, note that the observed CVFS proportions of BAU and Cash crops sum to more than 1, as some households farmed both types of crops in 2016-2017. By contrast, for reasons of tractability our model only allows agents to choose one or the other type of farming in any given cropping cycle, which may also lead us to underestimate the proportion of households continuing to farm BAU crops.

*Figure S3. Validation of ABM predicted distribution of livelihood strategies against CVFS data, 2017.*

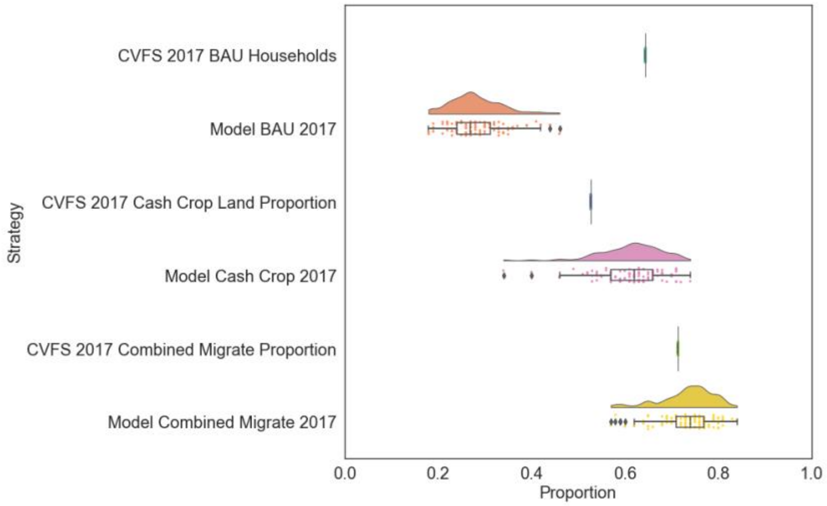


A closer examination of the distribution of farming households by the number of migrants per household indicates that the model may be capturing certain migration processes, while missing others. For example, while the model predictions closely match observed data regarding the number of households without migrants (model prediction: 26.6 percent; CVFS data: 28.6 percent), it underestimates the number of households sending only one migrant (model prediction: 2.0 percent; CVFS data: 28.2 percent) and overestimates the number of households sending two (29.6 vs. 16.3 percent) or three migrants (29.8 vs. 10.1 percent). This is likely due to the model's focus on labor migration as the main migration mechanism, in which each modelled household continues to send a migrant until the marginal productivity of migration (in terms of remittances/migrant) equals that of agriculture (in terms of agricultural revenues/household member). By contrast, many actual migrants from the Chitwan Valley may move for marriage, education or other life course transitions[22] where it may be more likely to have only one migrant per household.

*Figure S4. Visual comparison of the model and CVFS data.*

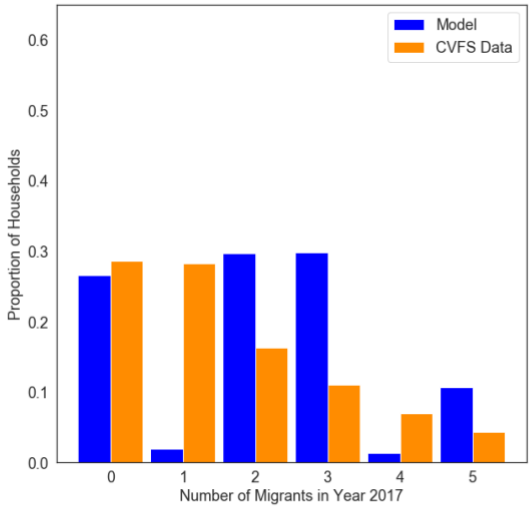


As the ABM includes both local (i.e., Nepal and India) and overseas (all other countries) migration destinations, we can also validate whether the model accurately captures the relative proportion of migrants to these two channels. As described in section SI 3.2, we use Nepal-wide data from Shrestha[19] to parameterize the relative migration costs, expected incomes, and standard deviations of remittances for these two destinations. Therefore, a good test is to assess how closely the model's predicted proportions

compare to the data reported in this study (Figure S4). Indeed, we find a close match: by 2017, the ABM predicts that 45.5 (95% CI: 44.3-46.6) percent of migrants use the local channel, while 54.5 percent (95% CI: 53.4-55.7) use the overseas channel. Shrestha[19] reports that 41 percent of Nepali migrants go to local destinations, and 59 percent go overseas. While these values do not lie in the 95 percent confidence interval of the model's predictions, the basic pattern that a slight majority of migrants use the international channel is captured by the model.

Finally, we turn to a pattern directly relevant to our research question: how does predicted migration behavior as a response to extreme drought compared to observed data? Here, we exploit the fact that 2014 represented an extreme drought year during both the dry and monsoonal cropping seasons in the Chitwan Valley area, with soil moisture conditions more than 3 standard deviations drier than the historical baseline for each season.[23] As 2014 falls within the CVFS timeline, we can calculate the net change in the number of migrants in the year after the drought (2015, roughly Nepali year 2072) vs. the year preceding the drought (2013, roughly Nepali year 2070). To generate a comparable set of model predictions, we re-run the ABM for the years 2007-2015, and implement an extreme drought for the 2014 crop cycles (time steps 14 and 15). We then similarly calculate the difference in the number of migrants between 2013 and 2015 to assess the net migration change during the drought. Note that pre-drought migration rates are not stationary in either the CVFS data or ABM: in each, there is a general increasing migration trend from 2007 to roughly 2015, even in the absence of drought. To isolate drought-induced migration (or immobility), we calculate a ratio of the proportional change in migrants from 2013-2015 to a baseline change from 2012-2013, as follows:

$$ratio = \frac{(m_{2015} - m_{2013}) \,/\, m_{2013}}{(m_{2013} - m_{2012}) \,/\, m_{2012}}$$

where $m_t$ indicates the number of migrants in year *t*. A ratio greater than 1 indicates that the annual proportional increase in migration rate was higher following the drought than before it; a ratio less than 1 indicates that the drought year attenuated the baseline increasing trend in migration.

The CVFS data indicates that there was a 19.7 percent increase in outmigration between the year preceding the drought (Nepali year 2070, roughly 2013) and the year following the drought (Nepali year 2072, roughly 2015). By contrast, in the two years preceding the drought (Nepali years 2068-2070, roughly 2011-2013), migration increased much more slowly, at 7.01 percent. The ratio of migration increases is 2.81, indicating that the annual increase in migration rate was 2.8 times higher in the years surrounding the drought, compared to the baseline years preceding it. The ABM captures a similar increase: the migration rate increases by an average of 15.3 percent in the years surrounding the drought, and 11.0 percent in the years preceding it. The ratio (1.39) is greater than 1, indicating that the ABM also captures an accelerating migration rate during extreme drought. However, it is smaller than the ratio from the CVFS data, indicating that the ABM may not be fully capturing the effect of drought on outmigration. This may especially be the case for short-term rural-rural migration, for example to work on another farm in a less drought-exposed area, which the ABM does not include as a potential livelihood option.

## 4 Supplementary References


1. The World Bank. World Bank Open Data | Data. https://data.worldbank.org/ (2022).
2. Otto, F. E. L. *et al.* Climate change increased rainfall associated with tropical cyclones hitting highly vulnerable communities in Madagascar, Mozambique & Malawi. 41 (2022).
3. Internal Displacement Monitoring Centre. *2021 Internal Displacement Index report*. https://www.internal-

displacement.org/sites/default/files/publications/documents/IDMC_Internal_Displacement_Index_Report_2021.pdf (2021).

4. Internal Displacement Monitoring Centre. Madagascar. *IDMC* https://www.internal-displacement.org/countries/madagascar (2022).
5. Langenbrunner, B. Water, water not everywhere. *Nat. Clim. Chang.* **11**, 650–650 (2021).
6. Liverman, D. M. Drought Impacts in Mexico: Climate, Agriculture, Technology, and Land Tenure in Sonora and Puebla. *Annals of the Association of American Geographers* **80**, 49–72 (1990).
7. Garip, F. On the move: Changing mechanisms of Mexico-US migration. *Princeton, NJ: Princeton University Press. doi* **10**, (2016).
8. Garip, F. & Asad, A. L. Network Effects in Mexico–U.S. Migration: Disentangling the Underlying Social Mechanisms. *American Behavioral Scientist* **60**, 1168–1193 (2016).
9. Feng, S., Krueger, A. B. & Oppenheimer, M. Linkages among climate change, crop yields and Mexico–US cross-border migration. *PNAS* **107**, 14257–14262 (2010).
10. Leyk, S., Runfola, D., Nawrotzki, R. J., Hunter, L. M. & Riosmena, F. Internal and International Mobility as Adaptation to Climatic Variability in Contemporary Mexico: Evidence from the Integration of Census and Satellite Data. *Population, Space and Place* **23**, e2047 (2017).
11. Nawrotzki, R. J., Hunter, L. M., Runfola, D. M. & Riosmena, F. Climate change as a migration driver from rural and urban Mexico. *Environ. Res. Lett.* **10**, 114023 (2015).
12. Karki, R. & Gurung, A. An overview of climate change and its impact on agriculture: a review from least developing country, Nepal. *International Journal of Ecosystem* **2**, 19–24 (2012).
13. MoFE. *Climate change scenarios for Nepal for National Adaptation Plan (NAP)*. http://mofe.gov.np/downloadfile/MOFE_2019_Climate%20change%20scenarios%20for%20Nepal_NAP_1562647620.pdf (2019).
14. Central Bureau of Statistics. *Nepal-Census-2011-Vol1.pdf*. https://unstats.un.org/unsd/demographic-social/census/documents/Nepal/Nepal-Census-2011-Vol1.pdf (2012).
15. MoALMC. *Impact of climate finance in agriculture on the poor*. https://reliefweb.int/sites/reliefweb.int/files/resources/UNDP_NP-Impact-of-Climate-Change-Finance-in-Agriculture-on-the-Poor.pdf (2018).

16. Ghimire, D. J., Axinn, W. G. & Bhandari, P. Social change, out-migration, and exit from farming in Nepal. *Popul Environ* **42**, 302–324 (2021).
17. Central Bureau of Statistics. *Preliminary Report of National Population Census 2021*. https://docs.censusnepal.cbs.gov.np//Documents/9823adf7-8f40-4abb-880c-eb21bb65f2ce.pdf (2022).
18. Choquette-Levy, N., Wildemeersch, M., Oppenheimer, M. & Levin, S. A. Risk transfer policies and climate-induced immobility among smallholder farmers. *Nat. Clim. Chang.* **11**, 1046–1054 (2021).
19. Shrestha, M. *Push and Pull: A Study of International Migration from Nepal*. https://papers.ssrn.com/abstract=2913956 (2017).
20. Grimm, V. *et al.* Pattern-oriented modeling of agent-based complex systems: lessons from ecology. *science* **310**, 987–991 (2005).
21. Ghimire, D. J., Williams, N. E., Thornton, A., Young-DeMarco, L. & Bhandari, P. Strategies for origin-based surveying of international migrants. *Journal of Ethnic and Migration Studies* **45**, 1185–1206 (2019).
22. Williams, N. Education, gender, and migration in the context of social change. *Social science research* **38**, 883–896 (2009).
23. Vicente-Serrano, S. M., Beguería, S. & López-Moreno, J. I. A multiscalar drought index sensitive to global warming: the standardized precipitation evapotranspiration index. *Journal of climate* **23**, 1696–1718 (2010).
